# Scaphoid Malunion and the Risk of Degenerative Arthritic Wrist: A Narrative Review of Biomechanics and Clinical Outcomes

**DOI:** 10.7759/cureus.103610

**Published:** 2026-02-14

**Authors:** Jae Jun Nam, Galen R Cummings

**Affiliations:** 1 Hand Surgery, Christine M. Kleinert Institute for Hand and Microsurgery, Louisville, USA

**Keywords:** degenerative wrist arthritis, scaphoid fracture, scaphoid malunion, scaphoid osteotomy, snac wrist

## Abstract

Scaphoid malunion is a common radiographic outcome following scaphoid fracture healing and has traditionally been assumed to predispose patients to degenerative wrist arthritis, often by analogy to scaphoid nonunion advanced collapse (SNAC). However, the clinical relevance and natural history of scaphoid malunion remain incompletely defined. This narrative review synthesizes the literature addressing radiographic definitions, biomechanical consequences, clinical outcomes, and reported management strategies for scaphoid malunion, with particular emphasis on its proposed relationship to SNAC wrist. Available studies demonstrate substantial heterogeneity in radiographic criteria used to define malunion, most commonly involving intrascaphoid angle, humpback deformity, and scaphoid length or alignment. Biomechanical investigations show altered wrist kinematics and load distribution in malunion; however, preservation of osseous continuity distinguishes malunion fundamentally from nonunion and does not reproduce the carpal instability patterns characteristic of SNAC. Importantly, clinical outcome studies do not consistently demonstrate increased pain, worse functional outcomes, or higher rates of secondary intervention in patients with scaphoid malunion compared with well-aligned unions, and progression to symptomatic degenerative arthritis is not reliably observed. Reported treatment strategies range from observation to corrective osteotomy and salvage procedures, with outcomes largely determined by patient symptoms and clinical context rather than radiographic deformity alone. Overall, current evidence suggests that scaphoid malunion represents a heterogeneous condition in which radiographic deformity and biomechanical alteration do not reliably predict clinical deterioration or inevitable progression to SNAC wrist, supporting a cautious, individualized, and symptom-driven approach to management.

## Introduction and background

Scaphoid fractures are the most common carpal fractures and are associated with well-recognized complications related to nonunion, altered carpal biomechanics, and degenerative wrist arthritis. The natural history of chronic scaphoid nonunion has been extensively described and may culminate in scaphoid nonunion advanced collapse (SNAC) wrist, a predictable pattern of progressive radiocarpal and midcarpal degeneration driven by persistent osseous discontinuity and carpal instability.

In contrast, the clinical significance and natural history of scaphoid malunion remain incompletely understood. Scaphoid malunion refers to the healing of a scaphoid fracture in a non-anatomic alignment, resulting in residual deformity of scaphoid morphology or carpal alignment despite osseous union. Scaphoid fractures are most commonly seen in young, active adults and represent the most frequent carpal fracture, with malunion occurring in a subset of patients following both operative and nonoperative treatment. Biomechanical investigations have demonstrated that scaphoid malunion can alter wrist kinematics, joint contact pressures, and load distribution across the carpus [1,2]. However, these mechanical alterations do not consistently translate into adverse clinical outcomes [3]. Several clinical outcome studies have reported comparable pain, range of motion, strength, and patient-reported outcomes between malunited scaphoids and well-aligned unions, raising questions about the true prognostic significance of deformity alone.

Despite these findings, treatment recommendations for scaphoid malunion remain heterogeneous. Some authors advocate corrective osteotomy in selected cases based on theoretical biomechanical concerns [1,4], whereas others report satisfactory outcomes with observation in asymptomatic or minimally symptomatic patients [2,5]. This variability reflects a broader disconnect between biomechanical data suggesting altered wrist mechanics and clinical studies demonstrating mixed or minimal functional consequences.

Given this discordance, there remains uncertainty regarding when scaphoid malunion warrants intervention and whether deformity alone is sufficient to predict symptoms or degenerative progression. A clearer synthesis of the biomechanical and clinical outcome literature may help reconcile these findings and inform a more rational approach to management.

The purpose of this review is to integrate existing biomechanical evidence, clinical outcome data, and treatment recommendations related to scaphoid malunion, with particular emphasis on the roles of load transmission and carpal stability. By summarizing what is currently known and identifying gaps in the literature, this review aims to provide an evidence-informed framework for evaluating and managing patients with scaphoid malunion.

Literature search strategy

This narrative review was informed by a non-systematic literature search of PubMed/MEDLINE and Google Scholar. English-language studies published from database inception through June 2024 were considered. Search terms included combinations of “scaphoid malunion,” “scaphoid fracture,” “humpback deformity,” “intrascaphoid angle,” “wrist biomechanics,” and “clinical outcomes.” Additional relevant articles were identified through manual review of reference lists. Given the heterogeneity of available studies, a narrative synthesis approach was used rather than a formal systematic review.

Given substantial heterogeneity in radiographic definitions of malunion, outcome measures, follow-up duration, and study design, no formal quantitative synthesis or meta-analysis was planned or performed. Evidence was integrated through a structured qualitative comparison of radiographic, biomechanical, and clinical domains. Biomechanical models, retrospective clinical cohorts, and systematic reviews were interpreted within the context of their methodological limitations.

## Review

Definitions and radiographic characterization of scaphoid malunion

Scaphoid malunion refers to fracture healing with residual deformity of scaphoid morphology or alignment; however, there is no universally accepted definition in the literature. Reported criteria for malunion vary widely and include angular deformities, changes in scaphoid length or height, alterations in carpal alignment, and qualitative assessments of radioscaphoid degeneration. Commonly used parameters include the intrascaphoid angle, scaphoid height-to-length ratio, presence of a humpback deformity, and associated lunate alignment abnormalities such as dorsal intercalated segment instability (Table 1). Importantly, threshold values used to define malunion differ substantially between studies, and many parameters describe scaphoid morphology rather than wrist function.

**Table 1:** Definitions and Radiographic Characterization of Scaphoid Malunion in the Literature *CT: Computed tomography, *MRI: Magnetic resonance imaging

Parameter	Definition / Measurement	Thresholds Reported	Representative Studies	Limitations
Intrascaphoid Angle (ISA)	Angle between proximal and distal scaphoid axes on lateral radiograph or CT	>35°–45°	Lynch et al. (1997) [4], Gillette et al. (2017) [2], Xiao et al. (2023) [5]	Variable thresholds; weak correlation with symptoms
Scaphoid Height-to-Length Ratio (HLR)	Ratio of scaphoid height to longitudinal length on sagittal imaging	<0.65–0.70	Boe et al. (2019) [1], Xiao et al. (2023) [5]	Technique dependent; limited predictive value
Humpback Deformity	Flexion deformity of the scaphoid waist	Qualitative or angle-based	Boe et al. (2019) [1], Lynch et al. (1997) [4]	Lacks standardized measurement
Lunate Alignment (DISI)	Lunate dorsiflexion on lateral radiograph	>10°–15°	Gillette et al. (2017) [2], Xiao et al. (2023) [5]	Not universal in malunion cases
Carpal Height Ratio	Ratio of carpal height to third metacarpal length	Decreased values reported	Historical series	Nonspecific; influenced by multiple variables
Radioscaphoid Degeneration	Joint space narrowing, osteophyte formation	Qualitative	Seltser et al. (2020) [16]	Degeneration does not necessarily correlate with symptoms
CT-based Morphology	Three-dimensional scaphoid geometry and alignment	No consensus thresholds	Xiao et al. (2023) [5]	Limited clinical correlation
MRI Cartilage Status	Assessment of articular cartilage integrity	Qualitative	Rarely reported	Not routinely obtained in practice

The intrascaphoid angle (ISA) is one of the most frequently reported metrics and is defined as the angle between the longitudinal axes of the proximal and distal scaphoid segments on lateral radiographs or sagittal computed tomography [6]. Thresholds used to define malunion based on ISA vary widely, ranging from approximately 35° to 45° [2,4,5], reflecting the absence of a standardized cutoff and inconsistent correlation with clinical symptoms. The scaphoid height-to-length ratio (HLR) is another commonly cited parameter, intended to quantify scaphoid shortening or collapse on sagittal imaging [7]; reported abnormal thresholds generally range from less than 0.65 to 0.70, though measurement technique and imaging modality significantly influence reproducibility [1,5].

Humpback deformity refers to flexion angulation of the scaphoid waist and is variably defined using qualitative assessment or angular measurements, without a universally accepted quantitative threshold [1]. Lunate alignment abnormalities, particularly dorsal intercalated segment instability (DISI), are occasionally included as supportive findings, typically defined by lunate dorsiflexion exceeding 10°-15° on lateral radiographs, although such findings are not universally present in malunion cases [2,5]. Additional parameters, such as carpal height ratio, have been reported in historical series but lack specificity, as they are influenced by multiple carpal and radiocarpal variables [8].

More recently, three-dimensional CT-based morphologic assessments have been proposed to better characterize scaphoid geometry [9]; however, no consensus thresholds have been established, and clinical correlations remain limited [5]. Radiographic degenerative changes at the radioscaphoid joint and qualitative MRI-based cartilage assessment have also been described [10,11], though these findings do not reliably correlate with pain or functional impairment and are not routinely incorporated into diagnostic criteria [12]. Collectively, these parameters illustrate the heterogeneity of radiographic definitions used to describe scaphoid malunion and underscore the lack of a standardized, clinically predictive framework.

No single radiographic measure reliably predicts symptoms, functional impairment, or degenerative progression. This lack of standardization complicates comparison across studies and likely contributes to the inconsistent associations reported between scaphoid malunion and clinical outcomes. Recognition of this definitional heterogeneity is critical when interpreting both biomechanical and clinical outcome studies, as differences in patient selection and malunion characterization may significantly influence reported results.

Biomechanical consequences of scaphoid malunion

Biomechanical investigations have consistently demonstrated that scaphoid malunion alters wrist kinematics and load distribution across the carpus. Cadaveric experiments, computational modeling, and in vivo imaging studies have shown changes in scaphoid flexion, radioscaphoid joint contact mechanics, and coordination between the proximal and distal carpal rows [6,7,12,13]. These findings have raised concern that residual deformity following scaphoid fracture healing may predispose the wrist to abnormal loading patterns and, potentially, degenerative change.

Importantly, however, the biomechanical consequences of scaphoid malunion differ fundamentally from those observed in scaphoid nonunion. In nonunion, persistent osseous discontinuity prevents the scaphoid from functioning as a structural strut linking the proximal and distal carpal rows, resulting in disrupted force transmission, uncoupling of carpal motion, progressive instability, and the characteristic pattern of degeneration observed in scaphoid nonunion advanced collapse [14,15].

In contrast, scaphoid malunion represents a healed fracture in which osseous continuity is preserved. Although malunion alters scaphoid geometry, it maintains a continuous load-transmission pathway between the carpal rows. Comparative biomechanical studies demonstrate that while malunion may modify joint contact pressures, scaphoid orientation, and carpal kinematics, it does not consistently reproduce the instability patterns characteristic of nonunion [14]. Finite element and computational contact analyses further suggest that increasing deformity severity is associated with focal increases in radioscaphoid contact stress; however, these models are limited by assumptions regarding material properties and do not account for long-term biologic adaptation [13].

In vivo motion analysis using four-dimensional computed tomography has further clarified this distinction, demonstrating that scaphoid nonunion results in uncoupling of proximal and distal carpal row motion, whereas preservation of osseous continuity in malunion maintains coordinated carpal kinematics despite altered scaphoid geometry [12]. Load transmission studies reinforce this concept by showing that the intact scaphoid serves as a primary load-bearing strut across the wrist; preservation of this structural continuity in malunion likely mitigates mechanical failure despite deformity [15].

Collectively, these biomechanical data underscore an important distinction between deformity and instability (Table 2). While scaphoid malunion alters wrist biomechanics, altered mechanics do not necessarily equate to biomechanical failure. Preservation of osseous continuity appears to be the critical factor differentiating malunion from nonunion and may help explain the discrepancy between biomechanical concern and the generally favorable clinical outcomes reported in long-term follow-up studies.

**Table 2:** Biomechanical Studies Evaluating the Consequences of Scaphoid Malunion *CT: Computed tomography

Study Type	Model	Biomechanical Variable Assessed	Representative References	Key Findings
Computational contact studies	Finite element modeling	Radioscaphoid contact pressure	Zhang et al. (2020) [13]	Increasing malunion severity is associated with increased focal radioscaphoid contact stresses
Cadaveric kinematic studies	Cadaver wrists	Scaphoid flexion, carpal alignment	Bain et al. (1998) [7], Amadio et al. (1989) [6]	Humpback deformity alters scaphoid flexion and intercarpal kinematics
In vivo motion analysis	Dynamic CT (4D CT)	Carpal row coordination	de Roo et al. (2019) [12]	Scaphoid nonunion results in uncoupling of proximal and distal carpal rows, whereas preserved continuity maintains coordination
Comparative biomechanical studies	Cadaveric / imaging-based	Stability patterns	Oka et al. (2011) [14]	Malunion alters wrist mechanics but does not reproduce instability patterns observed in nonunion
Load transmission analyses	Cadaveric force studies	Force transfer across the wrist	Viegas et al. (1990) [15]	The scaphoid functions as a primary load-bearing strut in the intact wrist

Clinical outcomes of scaphoid malunion

Clinical outcome studies provide a more nuanced perspective on the significance of scaphoid malunion. Multiple retrospective cohorts and long-term follow-up studies have demonstrated that scaphoid malunion is not consistently associated with inferior pain scores, reduced range of motion, diminished strength, or worse patient-reported outcomes when compared with well-aligned scaphoid unions (Table 3).

**Table 3:** Clinical Outcome Studies of Scaphoid Malunion and Their Radiographic Definitions *ROM: Range of motion, *ISA: Intra-scaphoid angle, *HLR: Height-to-length ratio

Study	Study Design	Follow-up	Radiographic Definition of Malunion	Outcomes Evaluated	Key Clinical Findings
Gillette et al. (2017) [2]	Retrospective cohort	Long-term (>10 years)	Increased intrascaphoid angle and/or humpback deformity on radiographs	Pain, ROM, grip strength, secondary surgery	Scaphoid malunion not associated with inferior functional outcomes or higher rates of secondary surgery
Putnam et al. (2023) [3]	Retrospective cohort	Mid-term	Lateral intrascaphoid angle >45° and/or scaphoid height-to-length ratio >0.60	Pain scores, ROM, grip strength, reoperation	Malunion not associated with worse pain, motion, or need for additional intervention
Amadio et al.(1989) [6]	Retrospective series	Long-term	Radiographic humpback deformity and increased intrascaphoid angle	Radiographic findings, symptoms	Identified deformity without demonstrating predictable clinical deterioration
Xiao et al. (2023) [5]	Systematic review	Not applicable (heterogeneous follow-up across included studies)	Variable definitions (ISA, HLR, humpback deformity, carpal alignment)	Clinical outcomes across studies	No consistent association between malunion and inferior outcomes
Seltser et al. (2020) [16]	Scoping review	Not applicable (heterogeneous and inconsistently reported follow-up)	Heterogeneous radiographic criteria	Symptoms, arthritis, function	Natural history poorly defined; inconsistent progression to symptomatic arthritis

Several retrospective cohort studies form the foundation of the clinical outcome literature on scaphoid malunion. Long-term follow-up studies have demonstrated no significant association between radiographic scaphoid malunion and pain, wrist range of motion, grip strength, progression to arthritis, or need for secondary surgical intervention, suggesting that deformity alone does not reliably predict clinical deterioration [2]. Mid-term outcome analyses using defined radiographic thresholds for malunion similarly found no differences in patient-reported pain, wrist motion, grip strength, or reoperation rates when malunited scaphoids were compared with well-aligned unions [3]. Earlier retrospective series identified radiographic deformity following scaphoid fracture healing but did not demonstrate a consistent relationship between malunion and symptomatic or functional decline, providing early evidence that anatomic imperfection does not necessarily translate into clinical failure [6].

Higher-level evidence from systematic and scoping reviews further highlights the uncertainty surrounding the natural history of scaphoid malunion. Systematic review data demonstrate substantial heterogeneity in radiographic definitions of malunion and do not show a consistent association between malunion and inferior clinical outcomes across included studies [5]. Similarly, scoping reviews report that although radiographic degenerative changes may be observed over time, progression to symptomatic arthritis or scaphoid nonunion advanced collapse is not reliably demonstrated, and many patients with documented malunion remain asymptomatic or minimally symptomatic over long-term follow-up [16]. Collectively, these findings emphasize that radiographic deformity in isolation has limited prognostic value and must be interpreted in the context of patient symptoms and functional status.

Systematic and scoping reviews further underscore the uncertainty surrounding the natural history of scaphoid malunion. While some studies describe radiographic degenerative changes over time, consistent progression to symptomatic arthritis or SNAC-type collapse has not been demonstrated [2,5,16]. Importantly, many patients with radiographic malunion remain asymptomatic or minimally symptomatic over long-term follow-up [2].

Taken together, available clinical evidence suggests that scaphoid malunion represents a heterogeneous condition in which radiographic deformity does not reliably predict symptoms, functional impairment, or need for surgical intervention. These findings support a symptom-driven approach to management and emphasize the importance of correlating radiographic findings with clinical presentation.

Treatment strategies reported in the literature

Observation is the most commonly reported strategy for asymptomatic or minimally symptomatic scaphoid malunion. Several clinical series and review studies demonstrate satisfactory outcomes with nonoperative management in patients without progressive pain, instability, or functional limitation. Given the absence of consistent evidence linking malunion to predictable clinical deterioration, a conservative approach is widely considered appropriate in selected patients. This strategy emphasizes clinical monitoring rather than prophylactic intervention.

Corrective osteotomy has been described primarily for symptomatic scaphoid malunion in patients without established degenerative arthritis. Improvements in pain and wrist function following corrective osteotomy in selected cases have been reported, and subsequent reports have similarly demonstrated symptomatic benefit [4]. Proposed advantages include restoration of scaphoid length and alignment, improvement in carpal kinematics, and reduction of abnormal joint contact stresses.

However, corrective osteotomy is technically demanding and associated with potential morbidity, including nonunion, hardware complications, and stiffness [1,2,4]. Reported complication and failure rates vary widely across studies due to heterogeneous definitions of malunion, differing indications for intervention, and small sample sizes. Accordingly, reported percentages should be interpreted as approximate ranges rather than precise estimates. Moreover, no study has demonstrated that corrective osteotomy prevents the development of degenerative arthritis or progression to scaphoid nonunion advanced collapse (SNAC) wrist. Reported benefits are primarily related to symptom improvement rather than modification of long-term natural history. As such, corrective osteotomy is generally reserved for carefully selected, symptomatic patients after thorough discussion of risks and expected outcomes [17].

In the presence of established degenerative changes or persistent symptoms refractory to conservative measures, treatment strategies shift toward managing arthritis rather than correcting deformity, such as wrist arthrodesis, proximal row carpectomy, or other salvage procedures, depending on the pattern and severity of degeneration [18,19].

Across studies, patient-reported outcomes were assessed using heterogeneous instruments, most commonly the Disabilities of the Arm, Shoulder, and Hand (DASH) score and pain visual analog scales, with less frequent use of wrist-specific measures such as the Patient-Rated Wrist Evaluation (PRWE). This variability in outcome assessment further limits direct comparison and quantitative synthesis of reported results.

Collectively, available evidence supports a symptom-driven, individualized approach to treatment. Radiographic deformity alone is insufficient to mandate intervention, and the role of corrective osteotomy remains limited to selected symptomatic patients without advanced degenerative change (Table 4).

**Table 4:** Reported Management Approaches and Outcomes for Scaphoid Malunion *DASH: Disabilities of the Arm, Shoulder, and Hand questionnaire, *VAS: Visual Analog Scale, *PRWE: Patient-Rated Wrist Evaluation

Treatment Strategy	Typical Indications	Reported Outcomes / Benefits	Patient-Reported Outcome Measures (PROs) Used	Reported Complications	Representative Study
Observation / Nonoperative Management	Asymptomatic or minimally symptomatic malunion	Majority of patients remain asymptomatic or minimally symptomatic at mid- to long-term follow-up	DASH; pain VAS; subjective symptom reporting	Persistent or progressive symptoms prompting later intervention	Gillette et al. (2017) [2], Putnam et al. (2023) [3], Seltser et al. (2020) [16]
Corrective Scaphoid Osteotomy	Symptomatic malunion without established arthritis	Improvement in pain and wrist function reported in most patients; radiographic correction and union commonly achieved	DASH; pain VAS; grip strength-related functional scores	Nonunion or delayed union, hardware irritation or failure, postoperative stiffness, persistent pain	Boe et al. (2019) [1], Lynch et al. (1997) [4], Amadio et al. (1989) [6]
Corrective Osteotomy with Bone Grafting	Symptomatic deformity requiring length or angular correction	Restoration of scaphoid alignment with union achieved in most cases; functional improvement reported	DASH; pain VAS; subjective functional assessment	Graft nonunion or resorption, donor-site morbidity, stiffness	Lynch et al. (1997) [4],
					El-Karef et al. (2005) [17]
Salvage Procedures (limited wrist arthrodesis, proximal row carpectomy)	Symptomatic degenerative arthritis or failed prior treatment	Reliable pain relief with predictable trade-off of reduced motion or strength depending on procedure	DASH; PRWE; pain VAS	Loss of motion, nonunion (arthrodesis), reduced grip strength, progression of arthritis, potential need for further surgery	Watson et al. (1984) [18], Mulford et al. (2019) [19]

Discussion

This narrative review synthesizes the existing literature on scaphoid malunion, focusing on radiographic characterization, biomechanical consequences, clinical outcomes, and reported treatment strategies. Although scaphoid malunion has historically been viewed as a potential precursor to progressive wrist degeneration, largely by analogy to scaphoid nonunion, the available evidence does not consistently support this assumption [1,6].

Biomechanical investigations demonstrate that scaphoid malunion alters wrist kinematics and joint contact mechanics [6,7,13]. However, a key distinction between malunion and nonunion is preservation of osseous continuity. In scaphoid nonunion, loss of continuity disrupts the scaphoid’s role as a load-transmitting strut between the proximal and distal carpal rows, leading to carpal uncoupling and progressive collapse [15]. In contrast, while malunion results in altered geometry, existing biomechanical studies have not demonstrated reproducible instability patterns or predictable collapse analogous to scaphoid nonunion advanced collapse [14]. This distinction provides an important framework for interpreting biomechanical findings in a clinical context.

Clinical outcome studies further suggest that radiographic deformity alone has limited prognostic value. Across retrospective cohorts and long-term follow-up studies, scaphoid malunion has not been consistently associated with worse pain, reduced range of motion, diminished strength, or increased need for secondary intervention when compared with well-aligned unions [2,3]. Systematic and scoping reviews similarly highlight the absence of a clearly defined natural history, with many malunions remaining asymptomatic or minimally symptomatic over time [16]. Importantly, wide variability exists in how scaphoid malunion is defined radiographically, and outcome assessment is inconsistent across studies, limiting direct comparison and quantitative synthesis.

Reported treatment strategies reflect this heterogeneity. Nonoperative management is commonly described for asymptomatic or minimally symptomatic patients [1]. Corrective scaphoid osteotomy has been reported primarily in selected symptomatic cases without established degenerative change and may provide improvement in pain and function [4]. However, these procedures are technically demanding and associated with recognized morbidity, and there is no evidence that osteotomy alters the long-term risk of degenerative arthritis or SNAC wrist [1]. Salvage procedures, including limited wrist arthrodesis and proximal row carpectomy, are reported in the setting of established arthritis and address pain and function rather than scaphoid malunion itself.

Several limitations of the existing literature warrant consideration. Most studies are retrospective, involve small cohorts, and use heterogeneous inclusion criteria. Radiographic definitions of scaphoid malunion vary widely, and outcome assessment lacks standardization, with inconsistent use of validated patient-reported outcome measures and variable follow-up duration. Treatment studies frequently include mixed populations, including malunion, delayed union, and nonunion, further limiting malunion-specific conclusions. In addition, biomechanical studies rely on experimental models that may not fully reflect in vivo loading conditions or long-term adaptation. In addition, as a narrative review, this study is subject to potential selection and publication bias, as the included literature was identified through a non-systematic search, and positive or symptomatic outcomes may be overrepresented.

## Conclusions

Scaphoid malunion remains a commonly encountered radiographic finding following scaphoid fracture healing, yet its clinical implications are less definitive than traditionally assumed. While biomechanical studies demonstrate alterations in wrist kinematics and load transmission, the preservation of osseous continuity in malunion appears to mitigate the instability patterns that characterize scaphoid nonunion advanced collapse. Available clinical outcome data do not consistently associate scaphoid malunion with increased pain, functional impairment, or inevitable progression to degenerative wrist arthritis. The wide variability in radiographic definitions, outcome measures, and duration of follow-up across studies limits the ability to draw firm conclusions regarding its natural history. Consequently, radiographic deformity alone should be interpreted with caution and not be considered a reliable surrogate for clinical deterioration. Further prospective studies with standardized definitions and validated outcome measures are needed to better clarify the long-term significance of scaphoid malunion and to guide evidence-based management strategies.
